# Cultural identity as a cognitive buffer: enhancing creative self-efficacy in digital heritage education

**DOI:** 10.3389/fpsyg.2026.1798104

**Published:** 2026-07-06

**Authors:** Na Wang, Jingjing Li

**Affiliations:** 1 Department of Art Design and Education, Yantai Vocational College, Yantai, China; 2 Department of Basic Courses, Yantai Vocational College, Yantai, China

**Keywords:** cognitive load mitigation, creative self-efficacy, cultural identity, digital cultural heritage, instructional support, Mount Tai culture

## Abstract

**Introduction:**

In vocational digital heritage programs, technical complexity is often associated with a heavy cognitive load, which may hinder students from utilizing cultural capital. Drawing on Cognitive Load Theory and the cultural assets framework, this study aims to examine the associations among instructional support, cognitive load, cultural identity, and students’ creative self-efficacy (CSE).

**Methods:**

A quantitative cross-sectional design was employed. Data were collected from 333 vocational college students using an online survey. A moderated mediation model was evaluated using Structural Equation Modeling (SEM), and the cross-contextual stability of the theoretical model was assessed via multi-group analysis.

**Results:**

SEM analysis revealed that instructional support is positively associated with CSE, and this relationship is primarily mediated by perceived cognitive ease (lower cognitive load), with the indirect path accounting for 54.4% of the total relationship. Crucially, cultural identity serves as a significant moderator; the negative association between cognitive load and CSE is weaker among students with strong cultural connections. Multi-group analysis confirmed the consistency of this structural model across different cultural heritage topics.

**Discussion:**

These correlational findings support a “Load-Asset Balance” mechanism, suggesting that cultural identity functions as an active psychological resource rather than a mere background variable. Cultivating learners’ emotional connection to traditional culture appears as essential as technical scaffolding. Future digital heritage pedagogy should integrate cognitive load management with cultural engagement to support creative confidence.

## 1 Introduction

### 1.1 Background and motivation

Digital transformation has fundamentally reshaped the preservation and experience of traditional culture, as illustrated by initiatives such as the “Digital Palace Museum” and immersive virtual tourism. It has also created an urgent demand for vocational talent capable of bridging historical literacy with modern technical skills. This demand is particularly evident in China, where national cultural digitization policies and vocational education development plans have jointly emphasized the need to cultivate technically skilled talent for emerging cultural and creative industries (General Office of the CPC Central Committee and General Office of the State Council, 2022; Ministry of Education of the People’s Republic of China, 2024). Recent national internet development reports also suggest that digital media and online audiovisual services have become increasingly embedded in young people’s everyday cultural participation (China Internet Network Information Center, 2025).

Many institutions have launched programs in digital media arts and cultural preservation to equip students with these interdisciplinary skills. However, a significant gap remains between educational practices and industry requirements. Designing digital cultural heritage products is inherently complex. Learners face the dual challenge of navigating abstract cultural meanings—such as historical contexts—while simultaneously mastering intricate technical workflows like 3D modeling. This simultaneous processing imposes a substantial cognitive load on students (Sweller, 1988; Paas et al., 2003). As noted in recent studies, students frequently become overwhelmed by the combination of massive cultural information and complicated technical procedures, leading to mental exhaustion and low learning efficiency (Zou et al., 2025). This sustained cognitive load inevitably dampens their curiosity and confidence, undermining their creative self-efficacy (CSE)—the belief in their ability to produce innovative outcomes (Tierney and Farmer, 2002). Consequently, many students fall into a predicament where they “have learned the skills but lack the confidence to create” (Huang et al., 2022).

Comparable challenges have also been observed internationally, although their emphases differ across contexts. European digital heritage education often focuses on museum-based learning, virtual exhibitions, digital access, and the balance between historical authenticity and immersive experience (Champion, 2021; European Commission, 2021). In other cultural contexts, heritage education is increasingly associated with cultural diversity, ethical representation, community participation, and the preservation of local or Indigenous knowledge systems (UNESCO, 2022). Compared with these contexts, Chinese vocational education faces a distinctive dual pressure: students are expected to master applied digital-production skills while interpreting culturally dense symbolic systems such as Mount Tai culture. This makes China a meaningful context for examining how instructional support and cultural identity jointly shape students’ cognitive and creative processes.

Therefore, a critical challenge for current vocational education is to determine how to provide effective instructional support that scientifically reduces cognitive load and thereby fosters students’ creative confidence. While previous research has explored technology integration in heritage education (Ugap et al., 2025), few studies have deeply analyzed the underlying psychological mechanisms from the learner’s perspective. This study addresses this gap by providing empirical evidence for optimizing digital cultural heritage education.

### 1.2 Problem statement

Existing literature has examined the impact of learning environments on student innovation, yet most studies remain at a macro level. There is a lack of deep exploration into the internal psychological transmission mechanisms, specifically regarding three primary aspects:

*The underlying psychological mechanisms*: While it is generally accepted that instructional support aids learning, the specific pathway through which it translates into creative self-efficacy remains unclear. We propose that cognitive load serves as the critical mediator—essentially the “psychological gatekeeper.” We posit that effective instructional support acts as a ‘cognitive scaffold,’ reallocating limited cognitive resources: it liberates mental energy from extraneous processing, allowing it to be invested in germane creative generation (van Merriënboer et al., 2024; Zuo et al., 2023). This mediation pathway requires rigorous empirical testing.

*Individual differences in cultural processing*: Why do students react differently to the same high-load tasks? Social Cognitive Theory suggests that individual belief systems are crucial. In the context of heritage education, cultural identity—a student’s sense of pride and belonging toward their traditional culture—may be a critical, yet overlooked, psychological resource. Students with high cultural identity may possess greater psychological resilience, using this emotional connection as a buffer against the stress of cognitive load. However, the moderating effect of cultural identity on the relationship between cognitive load and creative self-efficacy has rarely been examined.

*Contextual universality*: Are these psychological mechanisms consistent across different cultural topics? Unlike general cultural subjects, ‘Mount Tai culture’ serves as a representative national symbol with deep historical roots. Such high-profile cultural IP may trigger distinct emotional responses and activate different levels of prior knowledge. It remains to be seen whether the proposed mechanism is universal or if it varies between specific, high-profile cultural IP contexts (like Mount Tai) and other general cultural contexts. This addresses the need for refined and generalizable teaching strategies.

### 1.3 Research purpose and research questions

To bridge these gaps, this study constructs a moderated mediation model based on Cognitive Load Theory and Social Cognitive Theory. Utilizing an “anchoring-generalization” survey strategy across vocational institutions nationwide, we aim to uncover the internal mechanisms of digital cultural heritage design learning. The specific research questions are as follows:

*RQ1*: Does instructional support have a direct positive impact on students’ creative self-efficacy?

*RQ2*: Does cognitive load play a mediating role between instructional support and creative self-efficacy?

*RQ3*: Does cultural identity moderate the relationship between cognitive load and creative self-efficacy?

*RQ4*: Are there significant differences in the proposed model between the “Mount Tai culture learning group” and the “other cultures learning group”?

### 1.4 Research significance

*Theoretical contributions*: This study advances existing research in three ways. First, it shifts digital heritage education research from a predominant focus on technology adoption, digital storytelling, immersive environments, and learning satisfaction to the learner’s internal psychological mechanism by identifying cognitive load as a key mediator between instructional support and creative self-efficacy. Second, it reconceptualizes cultural identity as an active psychological asset rather than a mere contextual background variable, showing how it buffers the negative influence of cognitive pressure. Third, by comparing the Mount Tai culture group with other cultural-learning groups, it provides cross-contextual evidence for the stability of the proposed mechanism. Together, these contributions extend Cognitive Load Theory into digital heritage education, integrate cognitive science with social psychology, and support the broader applicability of the “Load-Asset Balance” framework in heritage-based learning.

*Practical contributions*: From a pedagogical perspective, the findings offer an evidence-based framework for refining digital heritage curricula. The study emphasizes the importance of “load-reducing” instructional strategies, suggesting that educators should prioritize optimized resource presentation and technical scaffolding to minimize students’ mental depletion. More importantly, by identifying cultural identity as a critical protective factor, the research advocates for a pedagogical shift where fostering an emotional connection to traditional culture is treated as a strategic psychological asset to support resilience against learning challenges. Additionally, the specific insights derived from the Mount Tai culture case provide targeted references for developing high-value cultural IP curricula, thereby promoting a more scientific and differentiated approach to the high-quality development of digital heritage education in the vocational sector.

## 2 Literature review and research hypotheses

### 2.1 Scaffolding digital heritage learning: the role of instructional support

Instructional support, often conceptualized as instructional scaffolding within educational psychology, refers to the temporary, tailored assistance provided by educators or learning environments to enable learners to achieve tasks beyond their unassisted capabilities (Wood et al., 1976). In the specific context of digital cultural heritage design—a domain characterized by high technical and semantic complexity—instructional support functions as an essential external resource. Based on Belland et al. (2013) and the multidimensional framework by Sakiz et al. (2012), we operationalize instructional support into three core dimensions essential for digital heritage education:

*Content support*: The systematization of cultural knowledge and its integration with digital workflows, helping students navigate complex historical narratives.

*Resource support*: The provision of tangible assets, such as high-performance hardware, VR platforms, and curated digital libraries, which reduce the logistical burden of creation.

*Teacher support*: The emotional and instrumental guidance provided by instructors, particularly their ability to offer personalized feedback on technical and creative challenges.

Empirical evidence consistently links strong instructional support to enhanced learning outcomes. Well-structured content helps construct clear knowledge schemas (Tetzlaff et al., 2025), while effective teacher guidance serves as a source of social persuasion, boosting students’ confidence to innovate (Bandura, 1997).

Recent studies further suggest that scaffolding, adaptive instructional design, and well-designed immersive learning environments can improve learners’ confidence and performance in complex digital contexts by reducing uncertainty and supporting knowledge integration (Huang et al., 2022; Makransky and Petersen, 2021; Zuo et al., 2023; Tetzlaff et al., 2025). Therefore, in digital heritage learning, instructional support is expected to enhance students’ creative self-efficacy.

### 2.2 Cognitive load: the barrier to asset utilization

Cognitive Load Theory (CLT) posits that human working memory is limited, and learning is hindered if this capacity is overwhelmed (Sweller, 1988, 2022). In digital heritage learning, cognitive load acts as a significant barrier. It comprises:

*Intrinsic cognitive load*: Determined by the inherent complexity of the cultural content itself (e.g., deciphering the intricate meanings of Mount Tai’s inscriptions).

*Extraneous cognitive load*: Imposed by poor instructional design or cumbersome software interfaces—this is “ineffective” load that wastes mental resources.

*Germane cognitive load*: The “effective” load invested in schema construction and creative synthesis.

The goal of instructional design is to minimize extraneous load and manage intrinsic load to free up capacity for germane processing. A significant body of research, summarized in recent systematic reviews, demonstrates that applying established multimedia learning principles—such as ensuring coherence and signaling key information—is an effective strategy for achieving this cognitive optimization (Mayer, 2024; Çeken and Taşkın, 2022). We propose that without effective support, the high cognitive load inherent in digital tools creates a ‘cognitive barrier.’ This barrier blocks access to cultural knowledge, thereby inhibiting its transformation into creative output.

Recent studies on virtual reality, online learning, and cognitive-load design further confirm that signaling, segmentation, and adaptive scaffolding can reduce unnecessary mental effort and help learners allocate cognitive resources more efficiently (Albus et al., 2021; Skulmowski and Xu, 2022; Zuo et al., 2023). Accordingly, instructional support is expected to reduce students’ cognitive load in digital cultural heritage learning.

### 2.3 Creative self-efficacy: the outcome of successful integration

Creative self-efficacy (CSE) is defined as an individual’s belief in their ability to produce creative outcomes (Tierney and Farmer, 2002). Within Social Cognitive Theory (Bandura, 1997), CSE is essential for sustaining effort in the face of challenges. In the creative domain, students with high CSE are more resilient and willing to experiment with novel ideas (Hwang and Wu, 2025). CSE is cultivated through mastery experiences, vicarious modeling, and positive emotional states. We posit that by reducing cognitive load (a source of stress and negative affect), instructional support creates a facilitating psychological pathway, thereby fostering the self-belief required for creative expression.

Excessive cognitive load may consume the working-memory resources required for divergent thinking, design exploration, and creative problem solving. Since creativity involves uncertainty and exploratory risk-taking, learners need sufficient cognitive and emotional resources to maintain confidence in creative performance (Beghetto, 2021). Recent studies in design and technology-supported learning further suggest that high mental effort, cognitive overload, and anxiety can weaken learners’ creative cognition and creative self-efficacy (Hwang and Wu, 2025; Zou et al., 2025). Therefore, cognitive load is expected to negatively affect creative self-efficacy.

### 2.4 Cultural identity as a psychological asset

Cultural identity refers to an individual’s sense of belonging, pride, and commitment to their heritage (Yao, 2025). In this study, we reframe cultural identity not merely as a background variable, but as a potent psychological asset. According to the Conservation of Resources (COR) theory (Hobfoll, 1989), individuals strive to retain and protect resources, and the possession of strong internal resources (such as cultural pride) can buffer against the impact of stressors (such as cognitive load).

Zhang et al. (2025) highlighted that cultural emotional identity acts as an internal reservoir of motivation. When students feel a deep connection to the cultural content (e.g., Mount Tai culture), this connection generates positive emotional energy. This suggest that this “identity asset” may functions as a protective buffer: when students face the high cognitive demand of digital tools, their strong cultural pride provides the psychological resilience needed to persist, preventing their creative confidence from diminishing under pressure.

Recent research also indicates that identity-related emotions can enhance learners’ belongingness, motivation, and persistence in culturally meaningful activities (Yao, 2025; Zhang et al., 2025). From the perspective of Conservation of Resources theory, these identity-based emotions may function as internal psychological resources that help students cope with cognitive pressure. Therefore, cultural identity is expected to buffer the negative influence of cognitive load on creative self-efficacy.

### 2.5 Research model construction and hypotheses

Synthesizing the theoretical perspectives on instructional support, cognitive load, creative self-efficacy, and cultural identity discussed above, this study proposes an integrated moderated mediation model.

**Figure 1:**
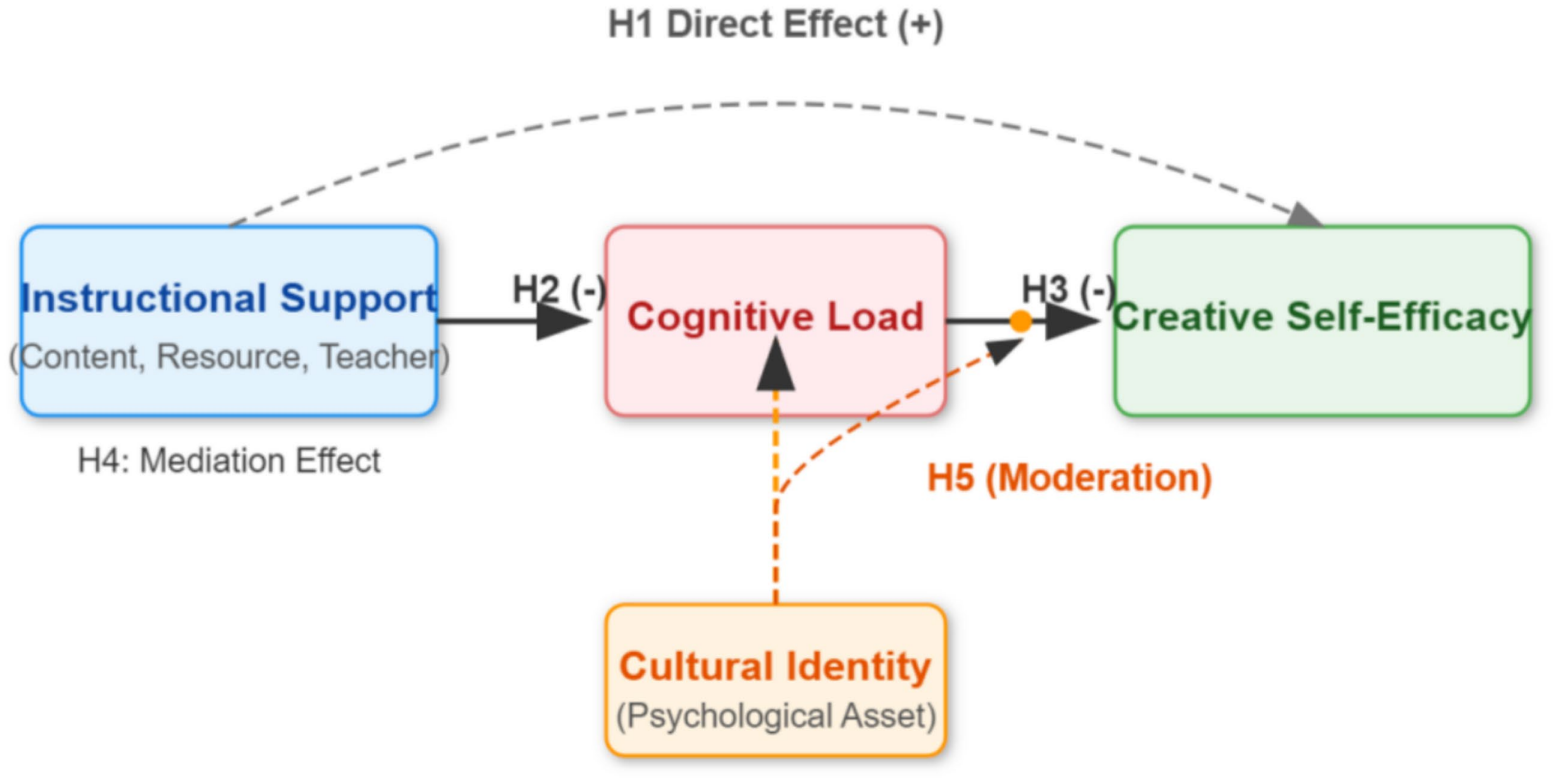
The conceptual model: mitigating load and harnessing assets. The model illustrates the moderated mediation mechanism where instructional support reduces cognitive load to enhance creative self-efficacy, while cultural identity serves as a crucial protective asset.

As illustrated in Figure 1, our conceptual model posits a distinct causal pathway:

(1) *The mitigation path*: Instructional support serves as the antecedent variable (Independent Variable), directly reducing cognitive load (Mediator).
(2) *The enhancement path*: A reduced cognitive load subsequently fosters creative self-efficacy (Dependent Variable).
(3) *The moderating path*: Significantly, cultural identity acts as a moderator (Social-Psychological Asset), buffering the negative link between load and efficacy.

**Figure 2:**
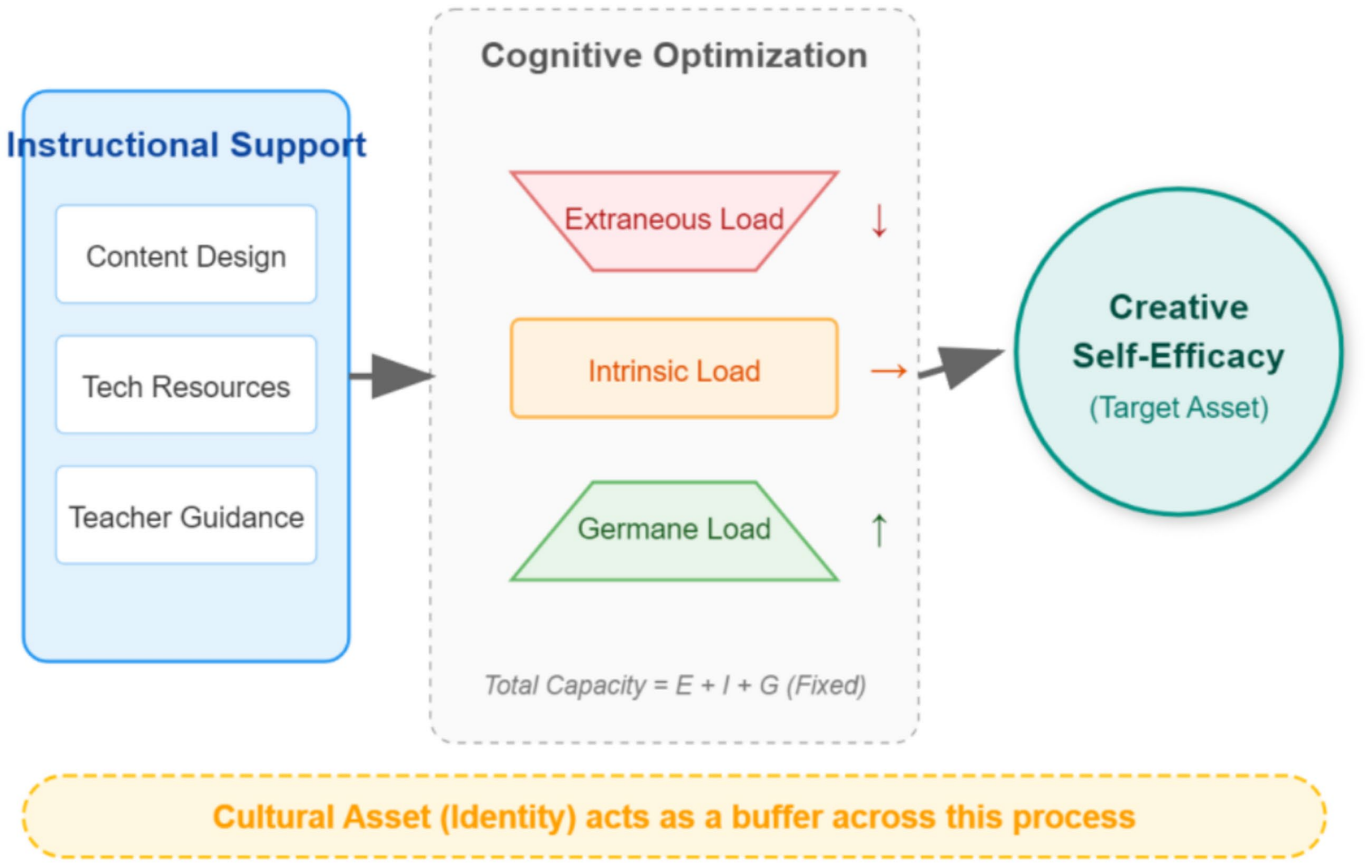
The cognitive resource optimization framework. This figure illustrates how the three dimensions of instructional support collectively optimize the allocation of cognitive resources—minimizing extraneous load and maximizing germane load—to ultimately achieve the target asset of creative self-efficacy, supported by the buffering role of cultural identity.

To further clarify how instructional support transforms cognitive resources, we developed a three-dimensional framework for cognitive resource optimization, as shown in Figure 2.

This framework visualizes the internal cognitive processing involved in digital heritage learning. Effective instructional support does not simply “lower difficulty” in a general sense; rather, it reallocates limited cognitive resources in three ways:

It reduces extraneous load (the red inverted trapezoid) by eliminating poor design;

It manages intrinsic load (the orange rectangle) found in complex cultural tasks;

Consequently, it frees up mental space to increase germane load (the green trapezoid), which is directly invested in creative thinking.

Based on the theoretical arguments above and the mechanisms illustrated in Figures 1, 2, the following hypotheses are formulated:

*H1*: Instructional support has a significant positive impact on creative self-efficacy.

*H2*: Instructional support has a significant negative impact on cognitive load.

*H3*: Cognitive load has a significant negative impact on creative self-efficacy.

The mediating role of cognitive load can be inferred from the relationship between instructional support and cognitive-resource allocation. Effective instructional support can reduce extraneous load and help learners devote more cognitive resources to schema construction, cultural interpretation, and creative synthesis (Skulmowski and Xu, 2022; Zuo et al., 2023; Mayer, 2024; van Merriënboer et al., 2024). Therefore, cognitive load is expected to serve as the psychological transmission mechanism through which instructional support influences creative self-efficacy.

*H4*: Cognitive load mediates the relationship between instructional support and creative self-efficacy.

*H5*: Cultural identity moderates the relationship between cognitive load and creative self-efficacy. Specifically, high cultural identity weakens the negative impact of cognitive load on creative self-efficacy.

Thus, based on the statistical relationships predicted in Figure 1 and the cognitive mechanisms explained in Figure 2, this study proceeds to empirical validation including the multi-group comparison corresponding to RQ4.

## 3 Research methods

### 3.1 Research design

This study employed a quantitative cross-sectional survey design to empirically test the proposed moderated mediation model. To balance specific case depth with generalizability, we implemented an “Anchoring-Generalization” strategy. Specifically, the survey was “anchored” in the context of the “Mount Tai Culture” project—a representative high-value cultural asset—to capture deep, context-specific experiences. Simultaneously, to broaden applicability, participants who had not engaged in this specific project were instructed to “generalize” their responses based on their experiences with other traditional Chinese cultural themes (e.g., the Forbidden City, regional intangible heritage). This dual-track design allows for a strong comparative verification of the “Cultural Asset Effect” across different contexts.

### 3.2 Participants and sampling procedure

The target population comprised students enrolled in vocational and technical colleges across China. Data were collected via the online platform “Wenjuanxing” using a controlled snowball sampling technique through academic and institutional networks. This approach was adopted because students with direct experience in digital cultural heritage design projects constitute a relatively specific and dispersed subgroup within vocational colleges, making it difficult to obtain a complete random sampling frame. To reduce potential selection bias, participants were recruited through multiple academic networks rather than a single class or institution, and eligibility was limited to students with relevant learning experience.

While non-probability sampling was employed, the recruited sample exhibits a balanced distribution across key demographic variables (e.g., gender, grade, and software proficiency, as detailed in Table 1), which helps mitigate potential selection bias and enhances the ecological validity of the findings. A total of 345 questionnaires were initially retrieved. Rigorous data cleaning was performed to ensure data quality:

**Table 1:** Demographic characteristics of the sample (*N* = 333).

Variable	Category	Frequency	Percentage (%)
Gender	Male	144	43.2
	Female	189	56.8
Grade	First year	121	36.3
	Second year	136	40.8
	Third year	61	18.3
	Other	15	4.6
Major	Design	112	33.6
	Computer science	98	29.4
	Cultural management	86	25.8
	Other	37	11.2
Experience level	No foundation/novice	167	50.2
	Experienced/advanced	166	49.8

*Exclusion criteria*: We excluded 5 respondents characterized by unrealistically short completion times (<60 s), suggesting insufficient cognitive processing. Additionally, 7 respondents who failed the attention check item (CL5: “Please select ‘Disagree’ for this item”) and those exhibiting obvious straight-lining response patterns were removed.

*Final sample*: Consequently, 333 valid samples were retained, yielding an effective response rate of 96.5%.

Based on Question 1 (“Select the option that best fits your learning experience”), the sample was stratified into two distinct groups:

*Group A (Mount Tai Culture Learning Group)*: 173 participants (52.0%)—Students who specifically focused on Mount Tai culture projects.

*Group B (Other Cultures Learning Group)*: 160 participants (48.0%)—Students who focused on other traditional cultural themes.

As shown in Table 1, the sample demonstrates a balanced demographic distribution, providing a diverse and appropriate representative foundation for model testing.

### 3.3 Measurement instruments

All measurement scales were adapted from established instruments with validated psychometric properties. To ensure contextual relevance, items were refined to fit the nuances of digital cultural heritage design. All items were rated on a 5-point Likert scale (1 = Strongly Disagree to 5 = Strongly Agree).

*Instructional support (TS)*: Adapted from the *Perceived Teacher Affective Support Scale* by Sakiz et al. (2012). Recognizing the multidimensional nature of design education, we operationalized this construct into three dimensions: Content Support (curriculum clarity), Resource Support (software/hardware availability), and Teacher Support (guidance and feedback), comprising 5 core items. Sample item: “The courses I take systematically explain the core connotations of the cultural heritage being studied.”

*Cognitive load (CL)*: Adapted from Paas (1992). The scale comprises 6 valid items (excluding the attention check) measuring perceived mental effort and task complexity. Important Note on Scoring: To facilitate the interpretation of structural equation modeling—where we hypothesize that instructional support positively influences a “facilitating” cognitive process—this variable was reverse-coded for data analysis. Thus, in the reported results, higher scores represent lower perceived cognitive load (i.e., higher Cognitive Ease).

*Creative self-efficacy (CSE)*: Adapted from Tierney and Farmer’s (2002) classic scale. This 4-item instrument assesses students’ confidence in their creative capabilities. Sample item: “I believe I have the ability to creatively solve difficulties encountered in design.”

*Cultural identity (CI)*: Adapted from the *Teacher Cultural Identity Scale* by Zhang et al. (2025). Aligning with our theoretical framework of “Cultural Assets,” we specifically focused on the Cultural Emotional Identity dimension to capture the affective strength of the student’s bond with their heritage. This 4-item scale measures the sense of pride and belonging. Sample item: “Thinking about traditional Chinese culture fills me with a sense of pride and identification.”

The full list of measurement items is provided in Appendix A.

### 3.4 Data analysis strategy

Data analysis was conducted using SPSS 26.0 and AMOS 24.0, adhering to standard SEM reporting guidelines (Kline, 2023).

AMOS 24.0 was selected because this study was designed for theory testing rather than exploratory prediction. Since the proposed model was derived from Cognitive Load Theory, Social Cognitive Theory, and Conservation of Resources theory, covariance-based SEM was appropriate for evaluating both the measurement model and the structural model through global fit indices such as *χ*^2^/df, CFI, TLI, RMSEA, and SRMR. Compared with SmartPLS, which is more prediction-oriented, AMOS better fits the confirmatory purpose of this study; compared with LISREL, AMOS provides a more intuitive graphical interface and is widely used in educational psychology and behavioral research.

The analytical procedure followed four steps:

*Descriptive statistics & bias testing*: Means, standard deviations, and correlations were calculated. Harman’s single-factor test was employed to assess Common Method Bias (CMB).

*Measurement model validation*: Confirmatory Factor Analysis (CFA) was conducted to evaluate Construct Validity (model fit), Internal Consistency (Cronbach’s *α* > 0.7), Composite Reliability (CR > 0.7), and Convergent/Discriminant Validity (AVE > 0.5).

*Structural model testing*: A Structural Equation Model (SEM) was built to test the hypothesized pathways (H1-H3). Mediation effects (H4) and moderation effects (H5) were tested using the Bootstrap method with 5,000 resamples to generate bias-corrected confidence intervals (Igartua and Hayes, 2021).

*Multi-Group Analysis (MGA)*: To answer RQ4, we performed a Multi-Group SEM. By constraining and unconstraining paths between Group A (Mount Tai) and Group B (Other), we tested for significant chi-square differences (Δ*χ*^2^) to determine if the “Asset Effect” varies across cultural contexts.

## 4 Data analysis and results

### 4.1 Common method bias and descriptive statistics

*Common Method Bias (CMB)*: To address potential bias inherent in self-reported data, Harman’s single-factor test was conducted on all items (unrotated). The first factor explained only 28.64% of the total variance, well below the 40% threshold (Podsakoff et al., 2003). Thus, CMB is not a substantial concern in this study.

*Descriptive statistics*: As presented in Table 2, the means, standard deviations, and correlations align with our theoretical expectations.

*Crucial note on interpretation*: Cognitive Load (CL) was reverse-coded. Therefore, the significant positive correlation between Instructional Support and CL (*r = 0.58, p < 0.01*) indicates that stronger support is associated with higher cognitive ease (i.e., lower load).

Similarly, Creative Self-Efficacy (CSE) is positively correlated with Cultural Identity (*r = 0.55, p < 0.01*), hinting at the potential of identity as a supportive asset.

**Table 2:** Descriptive statistics and correlation matrix of variables (*N* = 333).

Variable	M	SD	1	2	3	4
1. Instructional support (TS)	3.65	0.81	1			
2. Cognitive load (CL, reversed)	3.52	0.76	0.58**	1		
3. Creative self-efficacy (CSE)	3.71	0.83	0.62**	0.71**	1	
4. Cultural identity (CI)	3.78	0.84	0.55**	0.65**	0.76**	1

M, mean; SD, standard deviation. **p < 0.01. Cognitive Load items were reverse-coded for analysis (higher value = lower load).

### 4.2 Measurement model testing

Confirmatory factor analysis (CFA) using AMOS 24.0 validated the four-factor measurement model (TS, CL, CSE, CI). The model demonstrated excellent fit indices: $\chi^{2}/df=1.84$ , CFI=0.96 , TLI=0.95 , RMSEA=0.05 , SRMR=0.04 . All fit indices met or exceeded recommended standards.

*Reliability and validity*: As detailed in Table 3:

**Table 3:** Measurement model reliability and validity analysis.

Latent variable	Cronbach’s *α*	CR	AVE
Instructional support (TS)	0.79	0.80	0.56
Cognitive load (CL)	0.82	0.83	0.54
Creative self-efficacy (CSE)	0.81	0.82	0.61
Cultural identity (CI)	0.84	0.85	0.65

*Reliability*: Cronbach’s *α* (0.79–0.84) and Composite Reliability (CR > 0.80) for all constructs confirmed high internal consistency.

*Convergent validity*: Average Variance Extracted (AVE) values were all above 0.50.

*Discriminant validity*: The square root of the AVE for each construct was greater than its inter-construct correlations, supporting distinctiveness.

### 4.3 Structural model and hypothesis testing

#### 4.3.1 Mediation effect testing (the mitigation path)

A structural equation model was constructed to test the direct and mediated pathways. The structural model fit was strong ( $\chi^{2}/df=1.92$ [ideal <3.0], CFI=0.95 [good ≥0.90], TLI=0.95 , RMSEA=0.05 [good <0.08], SRMR=0.04 ).

Structural equation model path analysis results are shown in Table 4.

**Table 4:** Structural equation model path coefficients and fit indices (*N* = 333).

Path	Hypothesis	*β*	SE	CR	*p*	Conclusion
TS → CL	H2	0.64	0.06	10.45	<0.001	Supported
CL → CSE	H3	0.48	0.07	7.32	<0.001	Supported
TS → CSE	H1	0.26	0.06	4.81	<0.001	Supported

*β*, standardized path coefficient; SE, standard error; CR, critical ratio. Cognitive load uses reverse scoring; positive *β* indicates instructional support reduces cognitive load (increases ease).

All path coefficients passed Bootstrap 5,000 resampling validation.

Path a (TS → CL): Instructional support exhibited a significant positive impact on Cognitive Ease (reversed CL) ( β=0.64,p<0.001 ), implying that strong support effectively reduces cognitive burden. Thus, H2 is supported.

Path b (CL → CSE): Cognitive load (reverse scored) has a significant positive impact on creative self-efficacy ( β=0.48,p<0.001 ). That is, lower cognitive load correlates with higher creative self-efficacy. H3 is supported.

Path c’ (TS → CSE): After controlling for the mediator, instructional support’s direct impact on creative self-efficacy remains significant ( β=0.26,p<0.001 ). H1 is supported.

Using bias-corrected Bootstrap method (5,000 samples) to test mediation effects, results are shown in Table 5.

**Table 5:** Mediation effect decomposition and significance testing.

Effect type	Path	Effect Value	95% CI	Proportion	Conclusion
Total effect	TS → CSE	0.57	[0.48, 0.66]	100%	Significant
Direct effect	TS → CSE (c’)	0.26	[0.15, 0.37]	45.6%	Significant
Indirect effect	TS → CL → CSE (a × b)	0.31	[0.22, 0.41]	54.4%	Significant

The 95% confidence interval for the indirect effect [0.22, 0.41] does not include 0, indicating a significant mediation effect (*p* < 0.001). The indirect effect accounts for 54.4% of the total effect, confirming that cognitive load plays a partial mediating role between instructional support and creative self-efficacy. H4 is supported.

#### 4.3.2 Moderation effect testing (the asset-protection pathway)

To test cultural identity’s (CI) moderating role (H5), we employed the PROCESS macro (Model 14). We introduced the interaction term (CL × CI) into the model to predict CSE. Results showed the interaction term’s path coefficient was significant ( β=0.16,p<0.01 ), indicating the moderation effect holds.

**Figure 3:**
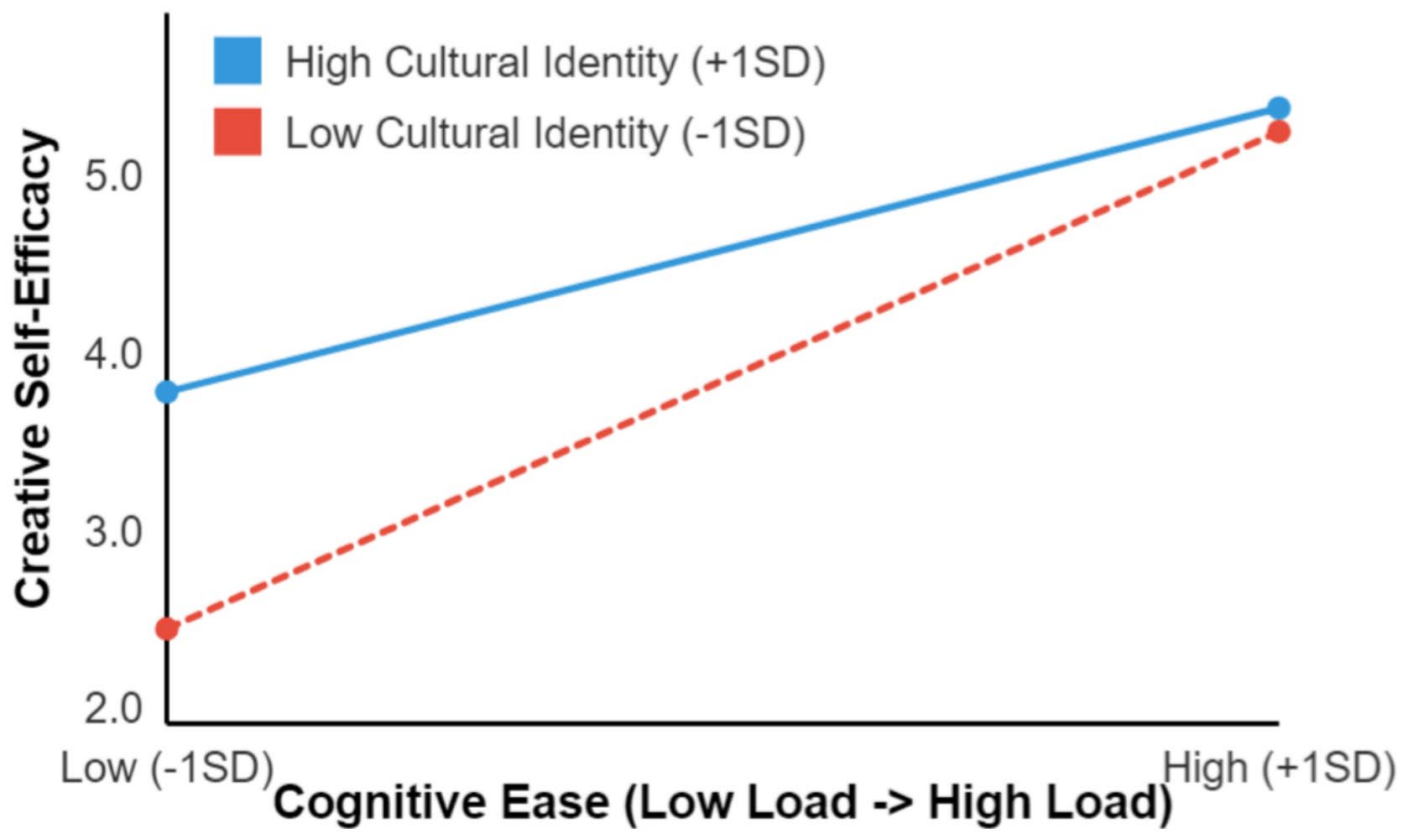
Simple slope analysis. The steeper slope for the low cultural identity group indicates their creative confidence is highly vulnerable to cognitive pressure. In contrast, the flatter slope for the high cultural identity group demonstrates resilience—their “cultural asset” protects their efficacy even under high load conditions.

**Figure 4:**
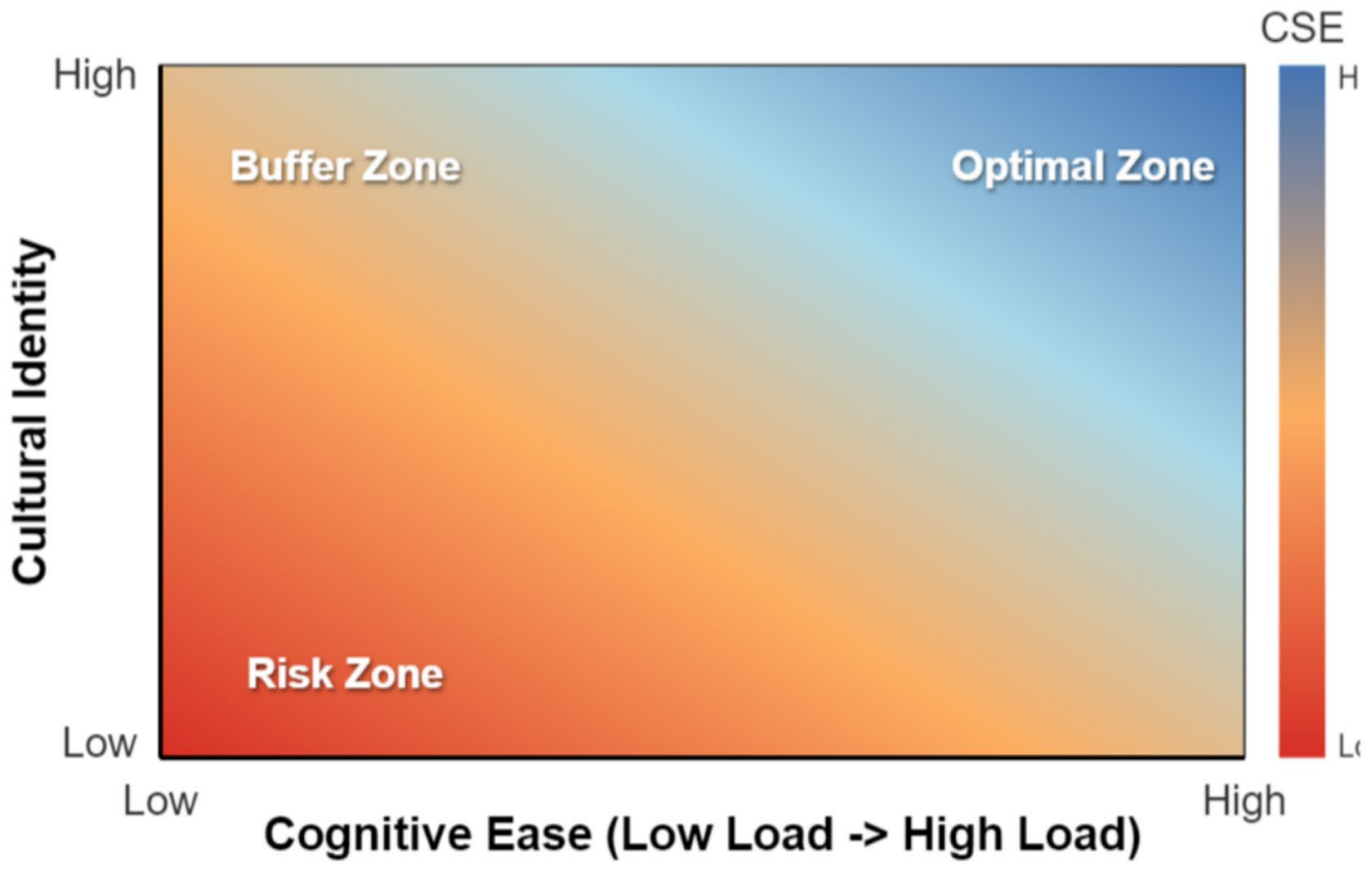
Heatmap of the moderation landscape. The “Buffer Zone” (upper right) visually confirms that high cultural identity allows students to maintain moderate-to-high creative self-efficacy despite experiencing significant cognitive friction.

To visualize this “Psychological Buffer Effect,” we conducted a simple slope analysis (see Figure 3) and a heatmap analysis (see Figure 4).

As shown in Figure 3, students with High Cultural Identity (Blue Line) show a flatter slope ( β=0.35,p<0.001 ). This indicates resilience: even when the task becomes cognitively demanding (low Ease), their creative confidence does not plummet. Conversely, students with Low Cultural Identity (Red Line) exhibit a steeper decline ( β=0.61,p<0.001 ), showing high vulnerability to stress. This empirical evidence confirms that cultural identity functions as a protective asset. H5 is supported.

Interpretation of Figure 4:

The heatmap reveals three distinct zones that tell a compelling story of asset utilization:

Risk Zone: (Low Identity + High Load) → Lowest Efficacy.

Buffer Zone: (High Identity + High Load) → Moderate Efficacy. Here, the “asset” of identity actively counteracts the “deficit” of load.

*Optimal zone*: (High Identity + Low Load) → Highest Efficacy.

Ideally, education should aim for the “Optimal Zone,” but the “Buffer Zone” proves that cultural assets are critical safeguards when technical difficulties are unavoidable.

### 4.4 Multi-group analysis results (RQ4)

To explore whether the model differs between the “Mount Tai culture group” (Group A) and the “other cultures learning group” (Group B), this study conducted multi-group analysis. We first established a baseline model (M1) with completely free path coefficients, then established a constrained model (M2) where all structural path coefficients were constrained to be equal across groups. We determined whether group differences exist by comparing chi-square value differences between the two models.

Results shown in Table 6 indicate that the chi-square value difference between the constrained model (M2) and the baseline model (M1) is not significant ( $\Delta \chi^{2}\left(4\right)=6.21,p=0.18>0.05$ ).

**Table 6:** Multi-group analysis model comparison.

Model	*χ* ^2^	df	Δ*χ*^2^	Δdf	*p*
M1: Unconstrained model	126.8	102	—	—	
M2: Constrained model	133.0	106	6.2	4	0.18

This result indicates that the core structural relationships among instructional support, cognitive load, creative self-efficacy, and cultural identity show no statistically significant differences between the two groups learning different cultural topics. This answers our research question RQ4: the psychological mechanism model constructed in this study demonstrates high cross-contextual generalizability, suggesting that the asset-protection mechanism is effective not only for specific high-value IPs like Mount Tai but also for broader cultural heritage contexts.

**Figure 5:**
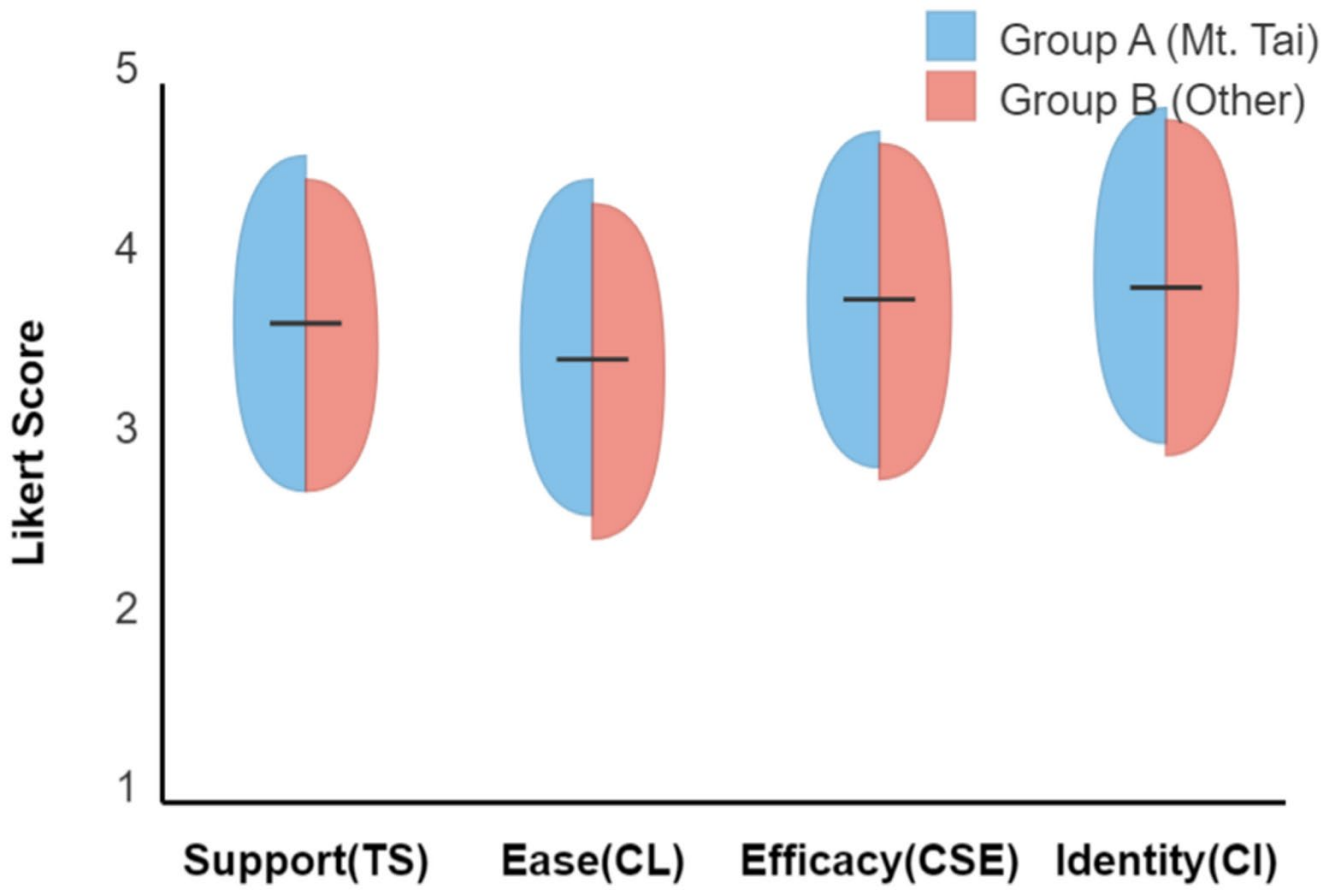
Comparative raincloud plot of key variables between groups. The high degree of visual overlap between Group A (Mount Tai culture, Blue) and Group B (other cultures, Red) across all constructs—instructional support, cognitive ease, creative self-efficacy, and cultural identity—visually confirms the statistical finding of cross-contextual universality (RQ4).

To visually examine these distributional similarities, we plotted a comparative Raincloud Plot (see Figure 5). As illustrated, the distribution patterns (clouds) and central tendencies (boxplots) for both Group A (Mount Tai) and Group B (Other) overlap substantially across all four primary variables.

## 5 Discussion

### 5.1 Summary of research findings

This study empirically validated a core psychological mechanism underlying digital cultural heritage design learning among 333 technical college students. The findings highlight three primary insights:

*The dominant mediating role of cognitive load*: While instructional support serves as an essential antecedent, its impact on efficacy is not direct. Instead, it operates primarily through the mediation of cognitive load. Support functions by transforming external complexity into internal cognitive ease. This “Load-Reduction Pathway” accounts for 54.4% (more than half) of the total effect, underscoring that cognitive management is not just a facilitator but the central driver of design education efficiency.

*Cultural identity as a protective resource*: Results provide compelling evidence that deep emotional attachment to traditional culture serves as an essential psychological buffer. For students possessing high “cultural assets” (identity), their creative confidence remains resilient even when facing significant cognitive pressure. This confirms that cultural emotion is not just a sentiment, but a functional resource.

*Cross-contextual stability*: The mechanism proved remarkably stable across different cultural contexts (Mount Tai vs. General). This suggests that the psychological interplay of “Support → Load → Efficacy” constitutes a universal fundamental mechanism in heritage education, transcending specific cultural IPs.

Overall, these findings are consistent with previous studies showing that instructional scaffolding and adaptive design can improve learning confidence by reducing unnecessary cognitive demands (Zuo et al., 2023; Mayer, 2024; van Merriënboer et al., 2024). They also extend cultural identity research by showing that identity is not only related to belonging and motivation, but also functions as a protective psychological resource in cognitively demanding digital heritage tasks (Yao, 2025; Zhang et al., 2025). Thus, the results are broadly consistent with prior research and further integrate cognitive-load reduction and cultural-identity activation into a unified explanatory model.

### 5.2 Theoretical implications

The study offers three significant theoretical contributions, particularly to the discourse on harnessing cultural assets in education:

One major contribution involves introducing the “Load-Asset Balance Model” within the context of digital heritage education. By integrating Cognitive Load Theory (CLT) with Social Cognitive Theory, we demonstrate that successful learning is a dynamic equilibrium: reducing the “deficit” of cognitive load (via support) while simultaneously leveraging the “asset” of cultural identity (Sweller, 2022; Bandura, 1997). This extends CLT from STEM fields into the culturally rich, emotionally charged domain of digital humanities (Zou et al., 2025).

Furthermore, the study redefines the role of culture in cognitive models. Traditional educational psychology often treats cultural background as a contextual variable. Our findings, however, elevate Cultural Identity to the status of a “Psychological Asset” (Hobfoll, 1989). By proving its moderating effect, we provide empirical support for integrating “hot” emotional variables into “cold” cognitive processing models (Igartua and Hayes, 2021).

Moreover, the confirmation of cross-contextual stability through Multi-Group Analysis establishes the “S-L-E” (Support-Load-Efficacy) mechanism as a content-independent cognitive architecture. The non-significant difference between the representative “Mount Tai” group and the general cultural group reveals a critical insight: the psychological pathway of learning digital heritage is isomorphic across different cultural themes. This suggests that the ‘Support–Load–Identity’ dynamic is not merely a situational reaction triggered by high-profile cultural IP, but a scalable pedagogical logic applicable to diverse cultural domains, from regional intangible heritage to world-renowned historical sites (Tetzlaff et al., 2025).

### 5.3 Practical implications

Guided by the “Load-Reduction” and “Asset-Activation” principles, we propose actionable strategies for educators and policymakers:

(1) Design for “Cognitive Friendliness” (The Load-Reduction Strategy)

Given that over 54% of the efficacy gain comes from reducing load, instructional design must prioritize load management (Mayer, 2024):

*Modular decomposition*: Break down complex heritage knowledge and software workflows into “micro-learning” units to lower intrinsic load.

*Curated resource pathways*: Replace open-ended information searching with curated “digital library navigators” to eliminate extraneous load.

*Just-in-time scaffolding*: Use AI or peer support to provide immediate technical help at bottleneck moments, preserving mental resources for creative thinking.

(2) Activate “Cultural Assets” (The Asset-Enhancement Strategy)

Educators should treat students’ cultural pride not as a passive background but as an active fuel:

*Value leadership*: Start courses with “Grand Narratives” of national or local heritage to ignite the “identity engine” (Explicit Motivation).

*Emotional bridging*: Connect abstract history to students’ personal hometown memories, transforming them from “observers” to “inheritors.”

*Community rituals*: Use showcases and ceremonies to reinforce the social validation of cultural creation, strengthening the protective buffer of identity.

(3) Global transferability

Although grounded in the Chinese context (Mount Tai), these principles are valid for international vocational education. Whether teaching Indigenous art in Australia or Renaissance history in Italy, the core strategy remains: Reduce the cognitive friction of technology so the emotional power of culture can emerge effectively. This study offers a framework for any nation seeking to revitalize its heritage through digital education.

### 5.4 Limitations and future directions

Despite its contributions, this study has limitations:

*Causality*: The cross-sectional design precludes strict causal inference. Future research should employ longitudinal or experimental interventions (e.g., controlling load levels) to verify the directionality of effects.

*Sampling*: Although the controlled snowball sampling strategy helped reach students with relevant digital cultural heritage learning experience across different academic networks, it remains a non-probability sampling method and may involve self-selection bias. Therefore, the findings should be generalized with caution and cannot be claimed to fully represent all vocational students in China. Future research should employ stratified random sampling or cluster sampling across different provinces, institution types, majors, and curriculum models to further test the robustness and representativeness of the model.

*Variable scope*: We focused on Cultural Identity. Future models could incorporate other “personal assets” such as Digital Literacy or Heritage Mindfulness to build a more comprehensive model of learner resilience (Marsh, 2023).

*Technological frontier*: A promising direction is AI-Driven Scaffolding. Future research should explore how Generative AI can dynamically adjust instructional support in real-time based on a student’s detected cognitive load, creating a truly adaptive “smart heritage classroom.”

## 6 Conclusion

This study advances our understanding of digital cultural heritage education by empirically validating a “Load-Asset Balance Model.” Based on a sample of 333 students, we move beyond simply confirming the benefits of instructional support, instead revealing the precise psychological pathway through which it operates: by mitigating cognitive load, it liberates mental resources for creative endeavors. Crucially, statistical analysis reveals that this mediation pathway accounts for 54.4% of the total effect. This evidence demonstrates that reducing cognitive friction is the primary driver for fostering creative confidence in students.

More importantly, our findings elevate cultural identity from a mere background characteristic to a functional psychological asset, demonstrating its power as a cognitive buffer that sustains creative confidence under pressure. These findings are consistent with prior research and extend existing work by showing how cognitive-load reduction and cultural-identity activation jointly support creative confidence in digital heritage education. The cross-contextual stability of this mechanism suggests its universal applicability, offering a foundational theory for “Heritage-Based Learning” that transcends specific cultural contexts. Therefore, this research advocates for a critical pedagogical shift in vocational education: moving away from intuition-based teaching towards an evidence-driven strategy that is both “Cognitively Friendly” and “Emotionally Activating.” By implementing this dual-pronged approach, educators can empower a new generation of “Digital Artisans”—individuals who are not just skilled technicians, but confident cultural innovators poised to revitalize our shared heritage in the digital age.

## Data Availability

The original contributions presented in the study are included in the article/Supplementary material, further inquiries can be directed to the corresponding author.

## References

[ref1] Albus P. Vogt A. Seufert T. (2021). Signaling in virtual reality influences learning outcome and cognitive load. Comput. Educ. 166:104154. doi: 10.1016/j.compedu.2021.104154

[ref2] Bandura A. (1997). Self-Efficacy: The Exercise of Control, vol. 11 New York: Freeman.

[ref3] Beghetto R. A. (2021). There is no creativity without uncertainty: Dubito ergo creo. J Creativity 31:100005. doi: 10.1016/j.yjoc.2021.100005, 38826717

[ref4] Belland B. R. Kim C. Hannafin M. J. (2013). A framework for designing scaffolds that improve motivation and cognition. Educ. Psychol. 48, 243–270. doi: 10.1080/00461520.2013.838920, 24273351 PMC3827669

[ref5] Çeken B. Taşkın N. (2022). Multimedia learning principles in different learning environments: a systematic review. Smart Learn. Environ. 9:19. doi: 10.1186/s40561-022-00200-2

[ref6] Champion E. (2021). Virtual Heritage: A guide. London: Ubiquity Press.

[ref7] China Internet Network Information Center (2025) The 55th Statistical Report on China’s Internet Development. Available online at: https://www.cnnic.cn/n4/2025/0117/c88-11129.html (Accessed May 15, 2026).

[ref8] European Commission (2021) Commission recommendation (EU) 2021/1970 of 10 November 2021 on a common European data space for cultural heritage Off. J. Eur. Union L 401 5–16. Available online at: https://eur-lex.europa.eu/legal-content/EN/TXT/?uri=CELEX:32021H1970 (Accessed May 15, 2026).

[ref9] General Office of the CPC Central Committee and General Office of the State Council (2022) Opinions on Promoting the Implementation of the national Cultural Digitization Strategy Xinhua News Agency. Available online at: https://www.gov.cn/zhengce/2022-05/22/content_5691759.htm (Accessed May 15, 2026).

[ref10] Hobfoll S. E. (1989). Conservation of resources: a new attempt at conceptualizing stress. Am. Psychol. 44, 513–524. doi: 10.1037/0003-066X.44.3.513, 2648906

[ref11] Huang Y. Li H. Fong R. (2022) Reducing cognitive load in cultural heritage learning through adaptive instructional design J. Educ. Technol. Soc. 25 78–92. Available online at: https://www.jstor.org/stable/27032847 (Accessed December 10, 2025).

[ref12] Hwang Y. Wu Y. (2025). The influence of generative artificial intelligence on creative cognition of design students: a chain mediation model of self-efficacy and anxiety. Front. Psychol. 15:1455015. doi: 10.3389/fpsyg.2024.1455015, 39931512 PMC11808137

[ref13] Igartua J. J. Hayes A. F. (2021). Mediation, moderation, and conditional process analysis: concepts, computations, and some common confusions. Span. J. Psychol. 24:e49. doi: 10.1017/SJP.2021.46, 35923144

[ref14] Kline R. B. (2023). Principles and Practice of Structural Equation Modeling. New York: Guilford Publications.

[ref15] Makransky G. Petersen G. B. (2021). The cognitive affective model of immersive learning: a theoretical research-based model of learning in immersive virtual reality. Educ. Psychol. Rev. 33, 937–958. doi: 10.1007/s10648-020-09586-2

[ref16] Marsh H. W. (2023). Extending the reciprocal effects model of math self-concept and achievement: long-term implications for end-of-high-school, age-26 outcomes, and long-term expectations. J. Educ. Psychol. 115, 193–211. doi: 10.1037/edu0000750

[ref17] Mayer R. E. (2024). The past, present, and future of the cognitive theory of multimedia learning. Educ. Psychol. Rev. 36:8. doi: 10.1007/s10648-023-09842-1

[ref18] Ministry of Education of the People’s Republic of China. (2024). Statistical Bulletin on the Development of national Education in 2023. Ministry of Education of the People’s Republic of China. Available online at: http://www.moe.gov.cn/jyb_sjzl/sjzl_fztjgb/202410/t20241024_1159002.html (Accessed May 15, 2026).

[ref19] Paas F. G. (1992). Training strategies for attaining transfer of problem-solving skill in statistics: a cognitive-load approach. J. Educ. Psychol. 84:429. doi: 10.1037/0022-0663.84.4.429

[ref20] Paas F. Renkl A. Sweller J. (2003). Cognitive load theory and instructional design: recent developments. Educ. Psychol. 38, 1–4. doi: 10.1207/S15326985EP3801_1

[ref21] Podsakoff P. M. MacKenzie S. B. Lee J. Y. Podsakoff N. P. (2003). Common method biases in behavioral research: a critical review of the literature and recommended remedies. J. Appl. Psychol. 88, 879–903. doi: 10.1037/0021-9010.88.5.879, 14516251

[ref22] Sakiz G. Pape S. J. Hoy A. W. (2012). Does perceived teacher affective support matter for middle school students in mathematics classrooms? J. Sch. Psychol. 50, 235–255. doi: 10.1016/j.jsp.2011.10.005, 22386122

[ref23] Skulmowski A. Xu K. M. (2022). Understanding cognitive load in digital and online learning: a new perspective on extraneous cognitive load. Educ. Psychol. Rev. 34, 171–196. doi: 10.1007/s10648-021-09624-7

[ref24] Sweller J. (1988). Cognitive load during problem solving: effects on learning. Cogn. Sci. 12, 257–285. doi: 10.1016/0364-0213(88)90023-7

[ref25] Sweller J. (2022). The role of evolutionary psychology in our understanding of human cognition: consequences for cognitive load theory and instructional procedures. Educ. Psychol. Rev. 34, 2229–2241. doi: 10.1007/s10648-021-09647-0

[ref26] Tetzlaff L. Simonsmeier B. Peters T. Brod G. (2025). A cornerstone of adaptivity–a meta-analysis of the expertise reversal effect. Learn. Instr. 98:102142. doi: 10.1016/j.learninstruc.2025.102142

[ref27] Tierney P. Farmer S. M. (2002). Creative self-efficacy: its potential antecedents and relationship to creative performance. Acad. Manag. J. 45, 1137–1148. doi: 10.5465/3069429

[ref28] Ugap C. Yahaya W. A. W. Balakrishnan B. Hashim M. E. A. H. Tochinai F. Nasir S. M. (2025). Tech-infused narrative: a systematic review of digital storytelling in education. J. Adv. Res. Design 131, 1–16. doi: 10.37934/ard.131.1.116a

[ref29] UNESCO. (2022). Re|shaping Policies for Creativity: Addressing Culture as a Global public good. UNESCO. Available online at: https://unesdoc.unesco.org/ark:/48223/pf0000380474 (Accessed May 15, 2026).

[ref30] van Merriënboer J. J. Kirschner P. A. Frèrejean J. (2024). Ten Steps to complex Learning: A systematic Approach to four-Component Instructional design. New York: Routledge.

[ref31] Wood D. Bruner J. S. Ross G. (1976). The role of tutoring in problem solving. J. Child Psychol. Psychiatry 17, 89–100. doi: 10.1111/j.1469-7610.1976.tb00381.x, 932126

[ref32] Yao M. (2025). The study of the effects of digital media applications in cross-cultural communication in the construction of cultural identity. Acta Psychol. 258:105247. doi: 10.1016/j.actpsy.2025.105247, 40651229

[ref33] Zhang L. M. Zhang Y. J. Chen Y. X. (2025). Development and application of teachers' cultural identity scale. Global Educ. 54, 141–160. (in Chinese)

[ref34] Zou L. Zhang Z. Mavilidi M. Chen Y. Herold F. Ouwehand K. . (2025). The synergy of embodied cognition and cognitive load theory for optimized learning. Nat. Hum. Behav. 9, 877–885. doi: 10.1038/s41562-025-02152-2, 40119235

[ref35] Zuo M. Kong S. Ma Y. Hu Y. Xiao M. (2023). The effects of using scaffolding in online learning: a meta-analysis. Educ. Sci. 13:705. doi: 10.3390/educsci13070705

