# Supplementary material for: Cultural identity as a cognitive buffer: enhancing creative self-efficacy in digital heritage education
Source: Front Psychol. 2026 Jul 6;17:1798104. doi: 10.3389/fpsyg.2026.1798104 (PMC13381194; doi:10.3389/fpsyg.2026.1798104)
Supplement: Supplementary file 1 [file Data_Sheet_1.ZIP › Appendix_A_Questionnaire.docx]

**Appendix A: Survey on Digital Cultural Heritage Learning Experience**

**Description:** This questionnaire is designed to investigate students’ perceptions of instructional support, cognitive load, creative self-efficacy, and cultural identity during digital heritage design tasks.

**Introduction & Consent:**
Dear Student, this survey aims to understand your learning experience in “Digital Cultural Heritage Design”. The questionnaire is anonymous, and all data will be used solely for academic research. Participation is voluntary. (Estimated time: 3-5 minutes)

**Part 1: Demographic and Background Information**

**Q1. Gender:**
□ Male
□ Female

**Q2. Current Grade Level:**
□ Freshman (Year 1)
□ Sophomore (Year 2)
□ Junior (Year 3)
□ Other

**Q3. Major Category:**
□ Art & Design (e.g., Visual Communication, Digital Media)
□ Computer Science/Technology
□ Cultural Heritage Management
□ Other

**Q4. Learning Experience Grouping:**
*Please select the option that best fits your experience to direct your subsequent answers:*
□ **Group A (Mount Tai Culture):** I have participated in learning or design projects specifically themed around “Mount Tai Culture”.
□ **Group B (General Culture):** I have not studied Mount Tai culture, but I have participated in other traditional culture design projects (e.g., The Forbidden City, Dunhuang, Intangible Heritage).
□ **Group C (No Experience):** I have never participated in such learning. *(End of Survey)*

**Q5. Proficiency with Design Software (Ps, 3D Max, Unity, etc.):**
□ Novice
□ Beginner
□ Intermediate
□ Advanced

**Part 2: Perceived Instructional Support (Independent Variable)**
*Please rate your agreement with the following statements regarding the support you received from teachers and the school. (1 = Strongly Disagree, 5 = Strongly Agree)*

**TS1.** The course systematically explained the historical and cultural connotations of the heritage site (e.g., Mount Tai).
**TS2.** Teachers provided abundant digital resources (e.g., high-res images, 3D assets) to support our design.
**TS3.** When I encountered technical difficulties, I received timely and effective guidance from instructors.
**TS4.** The hardware facilities and software platforms provided by the school met the design requirements.
**TS5.** Overall, the instructional support I received was comprehensive and helpful.

**Part 3: Cognitive Load (Mediator)**
*Please rate the level of* ***difficulty and mental effort*** *you experienced. Note: A higher score indicates higher difficulty/stress. (1 = Very Low/Easy, 5 = Very High/Difficult)*

**CL1 (Intrinsic).** The cultural knowledge involved in this project was very complex and difficult to understand.
**CL2 (Intrinsic).** The design task involved too many elements, making it mentally demanding to process.
**CL3 (Extraneous).** The way the teacher explained the design steps was confusing.
**CL4 (Extraneous).** The learning materials provided were disorganized, requiring extra effort to sort out.
**CL5 (Attention Check).** To confirm you are reading carefully, please select “Disagree” (Option 2) for this item.
**CL6 (Extraneous).** The operation of the software tools was cumbersome and frustrating.
**CL7 (Overall).** Overall, completing this design task required a tremendous amount of mental effort.

**Part 4: Creative Self-Efficacy (Dependent Variable)**
*Please rate your confidence in your creative abilities. (1 = Strongly Disagree, 5 = Strongly Agree)*

**CSE1.** I am confident in my ability to generate novel design ideas.
**CSE2.** I believe I can creatively solve problems encountered during the design process.
**CSE3.** I can skillfully use digital technology to express my creative concepts.
**CSE4.** I am confident that my final design work reflects high creativity.

**Part 5: Cultural Identity (Moderator)**
*Please rate your personal feelings toward the traditional culture you studied. (1 = Strongly Disagree, 5 = Strongly Agree)*

**CI1.** I feel a strong sense of pride and belonging involves the cultural heritage (e.g., Mount Tai) I studied.
**CI2.** I see myself as an inheritor and protector of this excellent traditional culture.
**CI3.** I am willing to actively introduce this culture to others even without course requirements.
**CI4.** I feel a deep spiritual connection to the core values of traditional Chinese culture.

*(End of Questionnaire)*
